# Dysregulated Glucose Metabolism as a Therapeutic Target to Reduce Post-traumatic Epilepsy

**DOI:** 10.3389/fncel.2018.00350

**Published:** 2018-10-16

**Authors:** Jenny B. Koenig, Chris G. Dulla

**Affiliations:** Department of Neuroscience, Tufts University School of Medicine, Boston, MA, United States

**Keywords:** glucose, traumatic brain injury, epilepsy, post-traumatic epilepsy (PTE), glycolysis, glycolytic inhibitors

## Abstract

Traumatic brain injury (TBI) is a significant cause of disability worldwide and can lead to post-traumatic epilepsy. Multiple molecular, cellular, and network pathologies occur following injury which may contribute to epileptogenesis. Efforts to identify mechanisms of disease progression and biomarkers which predict clinical outcomes have focused heavily on metabolic changes. Advances in imaging approaches, combined with well-established biochemical methodologies, have revealed a complex landscape of metabolic changes that occur acutely after TBI and then evolve in the days to weeks after. Based on this rich clinical and preclinical data, combined with the success of metabolic therapies like the ketogenic diet in treating epilepsy, interest has grown in determining whether manipulating metabolic activity following TBI may have therapeutic value to prevent post-traumatic epileptogenesis. Here, we focus on changes in glucose utilization and glycolytic activity in the brain following TBI and during seizures. We review relevant literature and outline potential paths forward to utilize glycolytic inhibitors as a disease-modifying therapy for post-traumatic epilepsy.

## Introduction

While accounting for only 2% of the body’s weight, the human brain accounts for 20% of its energy utilization (Rolfe and Brown, 1997). In pathological states, such as following a brain injury or during a seizure, the brain’s energy usage is significantly disrupted. In this review, we explore brain metabolism and glucose utilization as therapeutic targets to prevent the pathophysiological changes that may cause epileptogenesis following brain injury.

The brain requires energy in the form of ATP to power its cellular processes. The ability of the brain to conduct electrical signals between cells requires a steep electrochemical gradient to be maintained across cellular membranes. Reestablishing the electrochemical gradient following synaptic activity accounts for ≈80% of the total brain energy costs (Alle et al., 2009), with action potential (AP) firing contributing to a smaller, but important, component of energy utilization. Active transport of neurotransmitter into presynaptic vesicles, as well as vesicle recycling (Rangaraju et al., 2014), also requires ATP. Thus, the brain has many energetically intensive tasks in addition to basic cellular functions.

**FIGURE 1:**
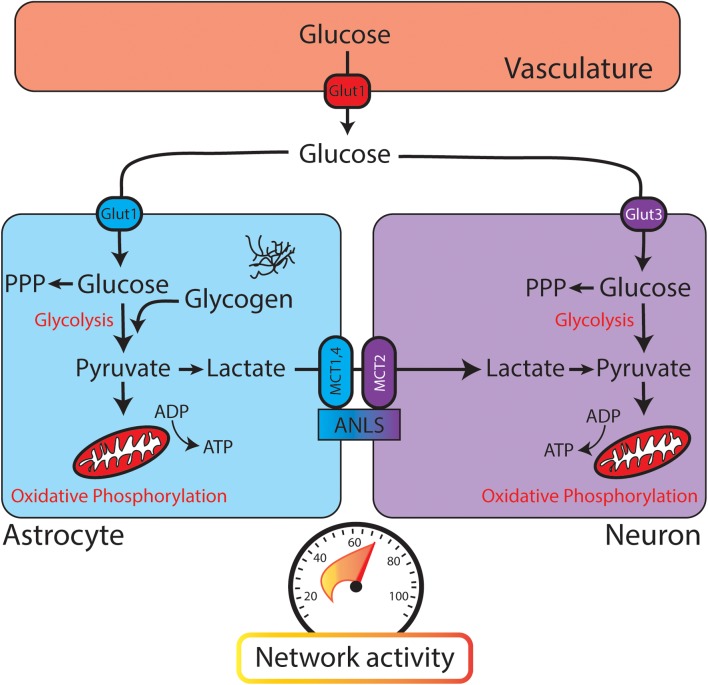
Glucose utilization in the brain relies on neuronal and astrocytic pathways. When glucose leaves the vasculature (red, **top**) it enters the brain parenchyma and is transported into astrocytes (blue) or neurons (purple). Both astrocytes and neurons contain glycolytic enzymes. The astrocyte-neuron lactate shuttle (ANLS) hypothesis posits that glycolysis is performed mainly in astrocytes, with lactate being delivered to neurons through monocarboxylate transporters (MCTs) for conversion to pyruvate and further metabolism through oxidative phosphorylation. PPP, pentose phosphate pathway. **(Bottom)** Speedometer represents the activity-dependent nature of brain metabolism. As network activity increases, glucose metabolism and energy consumption also increase.

The obligatory fuel of the brain is glucose, which is transported across the blood-brain-barrier by GLUT1 transporters (see **Figure 1**). The systemic delivery of multiple kinds of fuel (glucose, fructose, glycolytic end-products lactate and pyruvate, and ketone body β-hydroxybutyrate) results in an increase in extracellular glucose in the brain (Beland-Millar et al., 2017), suggesting that it is the preferred fuel. Only in extreme cases, such as during starvation or in the condition of the anticonvulsant ketogenic diet (discussed below), does the brain switch to utilizing a different energy source (ketone bodies) to generate ATP. In addition to glucose, the brain also requires oxygen. Both glucose and oxygen are delivered to the brain parenchyma through the vasculature, a process which is dynamically regulated by regionally- and temporally-specific changes in vasoconstriction and vasodilation. Thus, there is coupling between brain function and local vascular supply (first proposed by Roy and Sherrington in 1890), such that ultimately, energy delivery and utilization are activity-dependent processes.

Once a glucose molecule leaves the vasculature and enters the brain parenchyma, it is transported into cells for metabolism (through GLUT3 in neurons or GLUT1 in astrocytes). Canonically, glycolysis converts glucose to pyruvate, which is then converted to acetyl-CoA to enter the citric acid (TCA) cycle and provide substrates for oxidative phosphorylation (**Figure 3**). While oxidative phosphorylation ultimately generates the majority of the ATP in aerobic respiration, it also requires oxygen and is much slower than the glycolytic production of ATP. Thus, these two processes might fuel different aspects of brain function. As an alternative to glycolysis, glucose can be shuttled into the pentose phosphate pathway, which generates reduced NADPH and supports the production of glutathione. Glutathione provides an important defense mechanism against oxidative stress, as it serves as an electron donor in the detoxification of reactive oxygen species.

Different cell types in the brain utilize fuel differently. Neurons utilize 80–90% of the ATP of the brain, even though they account for ≈50% of the cells (Herculano-Houzel, 2014). A traditional view of brain glucose utilization poses astrocytes as a key metabolic support cell. In the “astrocyte-neuron lactate shuttle” (ANLS) model, glycolysis is performed in astrocytes and its product pyruvate is converted to lactate by lactate dehydrogenase (**Figure 1**). Lactate is then shuttled to neurons through monocarboxylate transporters (MCTs), converted back to pyruvate, and further metabolized through oxidative phosphorylation (Magistretti et al., 1993). This model is similar to energy utilization in other body tissues, where glycolysis and the TCA cycle are uncoupled at the level of lactate (Hui et al., 2017). While strong evidence supports the ANLS, there is robust evidence that glycolysis also occurs in neurons. Examination of transcriptional expression and proteomics data reveals the presence of components of the glycolytic pathway in both astrocytes and neurons (Yellen, 2018).

One of the challenges to understanding brain metabolism is effectively visualizing and integrating the movement of metabolites at the regional, cellular, and subcellular level. To understand large-scale changes, we can utilize whole brain imaging with functional MRI (fMRI) or positron emission tomography (PET) scanning. fMRI relies on the signal of oxy- versus deoxy-hemoglobin, where changes in the “BOLD” signal can occur with blood flow or metabolic flux. PET scanning, on the other hand, utilizes tracers to label blood, glucose, or oxygen. For example, using 18F-labeled 2-deoxyglucose (F-DG), one can visualize the accumulation of “glucose” in areas of the brain which require more fuel, and are thus transporting more glucose and F-DG into the brain parenchyma. We can also look at metabolic flux in brain slices from animal models or in culture, using metabolomic or molecular imaging of pH, NADH (Tantama et al., 2013; Hung and Yellen, 2014), ATP, or fluorescently labeled glucose (Bernardinelli et al., 2004). Using these micro and macro views of brain metabolism, we can begin to understand system-level metabolic activity in the healthy, injured, and epileptic brain.

**FIGURE 2:**
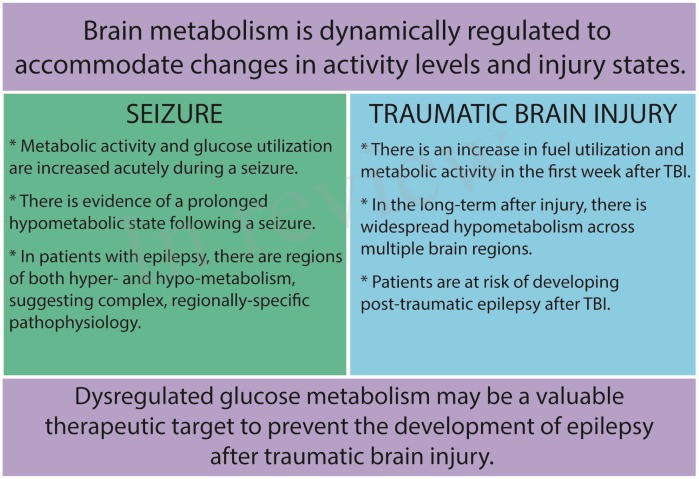
Key points.

A better understanding of brain fuel utilization at baseline and in pathological states may allow us to harness the therapeutic potential of metabolic targets. Our motivation for this approach is based on the robust anticonvulsant and neuroprotective effects of the ketogenic diet in humans (Rogawski et al., 2016), where very low-carbohydrate, high-fat intake causes a shift of brain fuel utilization away from glucose and toward ketone bodies. A number of exciting studies have attempted to harness the power of the ketogenic diet either by providing ketones as an exogenous energy supply or by inhibiting glycolysis using pharmacological approaches. These manipulations can have far-reaching effects on brain activity and neurological outcomes, well beyond acute changes in metabolic activity. Here, we will explore glycolytic inhibition as a potential therapeutic entry point in treating traumatic brain injury (TBI) and post-traumatic epilepsy (PTE; **Figure 2**).

## Metabolism Is Activity-Dependent

As the energy requirements of the brain change, fuel utilization can be upregulated to accommodate these requirements. For example, fMRI studies have shown that visual tasks result in increased cerebral blood flow and increased oxidative metabolism in the visual cortex to meet the metabolic needs of the region (Lin et al., 2010). However, there are many challenges in interpreting changes in cerebral blood flow and oxygen utilization at the level of the entire brain (Buxton, 2010), so *in vitro* experiments can aid in elucidating the mechanisms by which activity results in metabolic changes.

### Activity-Dependent Changes in Astrocytic Glucose Utilization

As shown in **Figure 1**, changes in network activity can affect a broad range of metabolic processes in both astrocytes and neurons. Astrocytes are poised to detect local changes in neuronal activity, and thus energetic need, in the brain. These cells have close interactions with synapses, and dynamically change their morphology in response to activity (Genoud et al., 2006). Astrocytes participate in glutamate uptake through transporters GLT-1 and GLAST, which themselves are upregulated by activity (Armbruster et al., 2016; Pinky et al., 2018). Through this mechanism, astrocytes sense local changes in excitatory activity based on changes in glutamate concentration.

It has been previously shown that glutamate uptake requires re-establishment of the sodium gradient across the membrane (as glutamate is co-transported with sodium), which results in a decrease of ATP in astrocytes (Magistretti and Chatton, 2005). Alongside this energetic need, glutamate transport stimulates increased glucose uptake and increased lactate release from astrocytes in culture (Pellerin and Magistretti, 1994). This study in particular provided key evidence for the ANLS hypothesis, which postulates that increases in activity result in astrocytic glycolysis and delivery of lactate to neurons as fuel. When glutamate uptake is reduced in GLT-1 or GLAST knockout mice, there is a decrease in glucose tracer uptake induced by unilateral whisker stimulation (Voutsinos-Porche et al., 2003). This lends further support, *in vivo*, that glutamate uptake in astrocytes plays a crucial role in glucose utilization. Additionally, astrocytes exhibit “metabolic waves” of fluorescently labeled glucose (2-NBD-glucose), where 2-NBD-glucose uptake parallels the spatial movement and kinetics of sodium transport into astrocytes (Bernardinelli et al., 2004). The neuroenergetic coupling of stimulation to glucose movement is inhibited with glutamate transport blocker TBOA, suggesting a necessary role for glutamate transporters in the activity-dependent effects on astrocytic glucose uptake. While elevated extracellular glutamate results in increases in glucose utilization in astrocytes, the sodium co-transport through glutamate transporters also acidifies mitochondrial pH such that oxidative phosphorylation is less effective (Azarias et al., 2011). This potential reduction in the efficiency of oxidative phosphorylation suggests a glutamate-uptake induced shift in astrocytic metabolism away from oxidative phosphorylation and toward glycolysis during periods of activity.

Other extracellular changes associated with neuronal activity also induce changes in astrocytic glycolysis. Increases in extracellular potassium concentration (such as following APs) result in increased deoxyglucose accumulation (Peng et al., 1994) and glycolytic activity in astrocytes (Bittner et al., 2011). This effect was found to be dependent on the Na^+^/HCO_3_^-^ cotransporter NBCe1 (Ruminot et al., 2011). Additionally, noradrenaline (via β2- and β3-adrenoreceptors) or arachidonic acid can stimulate glucose uptake in astrocytes (Yu et al., 1993; Hutchinson et al., 2007).

Astrocytes are also able to mobilize energy for utilization through glycogenolysis, where astrocytic glycogen stores are catabolized into single glucose units. Extracellular potassium has been shown to induce glycogenolysis in mouse cortical slices in a calcium-dependent manner (Hof et al., 1988). The breakdown of astrocytic glycogen stores can also be induced by adenosine, ATP, arachidonic acid, vasoactive intestinal peptide, and noradrenaline (Sorg and Magistretti, 1991; Sorg et al., 1995).

### Activity-Dependent Changes in Neuronal Glucose Utilization

While astrocytes provide metabolic support to neurons through the ANLS, neurons are also able to upregulate their own metabolic processes in response to activity. The ANLS model suggests that glycolysis occurs mainly in astrocytes, but there is significant evidence supporting that glycolysis takes place in neurons as well (reviewed in Ashrafi and Ryan, 2017). First, neurons have enriched expression of three isoforms of the rate-limiting enzyme of glycolysis (hexokinase) relative to astrocytes (Lundgaard et al., 2015). In addition, hexokinase-1 protein levels positively correlate with glucose uptake in neurons, suggesting that the cells with more hexokinase are more readily able to metabolize glucose through the glycolytic pathway. Finally, this study showed that functional activation with whisker stimulation increased *in vivo* glucose uptake in neurons, but not astrocytes, suggesting that neuronal glucose utilization is dynamic with activity state. Additionally, data mining of previously published transcriptomic and proteomic results show increased protein levels for many of the enzymes required for glycolysis in neurons relative to astrocytes (Yellen, 2018). Thus, neurons have the machinery required to utilize glucose through the glycolytic pathway.

As in astrocytic metabolism, activity also drives changes in neuronal metabolism. Synaptic activity requires ATP, however, in normal conditions, ATP levels stay constant in neurons following activity (Rangaraju et al., 2014). Blocking either the glycolytic pathway or oxidative phosphorylation results in a precipitous drop in ATP levels following synaptic activity in neuronal culture (Rangaraju et al., 2014). Taken together, these findings suggest that activity drives neuronal ATP production in order to maintain consistent ATP levels within the cell sufficient to meet the significant energy demands of the synaptic vesicle cycle.

The first step in upregulating fuel utilization in response to activity is to increase glucose delivery to the neurons. Synaptic transmission in cortical neuronal cultures has been shown to increase surface expression of GLUT3, a key glucose transporter in neurons, in an NMDA receptor-dependent manner (Ferreira et al., 2011). An additional glucose transporter, GLUT4, is also mobilized to the presynaptic surface as a result of synaptic firing (Ashrafi et al., 2017). Neurons with mutated GLUT4, which is defective in glucose transport, are unable to maintain synaptic vesicle recycling with activity (Ashrafi et al., 2017), suggesting a key role for fuel delivery to perform the energetically demanding processes at the synapse.

In the ANLS model, lactate would be delivered to neurons from astrocytes, instead of neurons taking up glucose directly. However, it has been shown that glucose uptake (not lactate utilization) is positively correlated with NMDA receptor-mediated activity in neurons (Bak et al., 2009). This finding was reproduced in hippocampal slices and *in vivo*, where activity caused an increase in glycolytic activity in neurons (measured by an increase in the cytosolic NADH:NAD^+^ ratio, which was not affected by blocking lactate delivery to neurons through MCTs; Diaz-Garcia et al., 2017).

While some studies have suggested that neurons rely on oxidative metabolism (Peng et al., 1994; Hall et al., 2012; Sobieski et al., 2017), glycolysis may provide faster replenishment of ATP in cases of high activity or energy stress. For example, recent work described the formation of a “glycolytic metabolon” at presynaptic sites, where glycolytic enzymes are locally concentrated to accommodate for energy demands at active synapses (Jang et al., 2016). This clustering allows for rapid, spatially controlled delivery of ATP to the synapses with increased energy need to fuel activity. Additionally, AP bursting in primary hippocampal culture results in a shift of the neuronal transcriptional profile away from oxidative phosphorylation and toward the glycolytic pathway (Bas-Orth et al., 2017). Thus, it seems that neurons may rely more heavily on oxidative phosphorylation at baseline, whereas a shift toward glycolysis during periods of high activity would allow rapid replenishment of energy to support synaptic function (resembling the distinction between “fast-twitch” muscle fibers which rely on glycolysis versus “slow-twitch” muscle fibers which utilize oxidative phosphorylation).

### Changes in Metabolism Feed Back to Affect Neuronal Activity

Altering neuronal metabolism can have direct effects on neuronal excitability and activity. Glycolytic inhibition with the pharmacologic inhibitor iodoacetic acid alters the shape of the neuronal AP, resulting in a smaller and broader presynaptic AP waveform (Lujan et al., 2016). Altering the availability of glucose can also have effects on broader network activity, such as reducing the power of gamma oscillations in hippocampal slices (Galow et al., 2014). These effects may be mediated through molecules which can sense local energy state and affect neuronal excitability. For example, K_ATP_ channels conduct potassium in response to decreased ATP concentration, and can thus directly modulate neuronal activity by altering membrane resistance (Tanner et al., 2011; Lemak et al., 2014; Lutas et al., 2014). Another example of metabolism feeding back to neuronal excitability is through adenosine receptors. Adenosine release, which can occur via equilibrative transporters when ATP consumption is high and ATP production is low, decreases excitatory synaptic transmission during hypoxia via A_1_ receptors, (Pearson et al., 2001). Conversely, adenosine can increase the intrinsic excitability of pyramidal neurons via A_2A_ receptors, (Rau et al., 2015). Understanding how metabolic manipulations can impact neuronal activity will provide novel tools for us to control network output in cases of pathological activation, such as following a brain injury or during a seizure.

## Glucose Utilization During Seizures

At the most basic level, metabolic activity increases acutely during a seizure or convulsion (Sacktor et al., 1966), with increased rates of both glucose and oxygen consumption (Wasterlain et al., 1993). Human imaging studies also show that glucose utilization is elevated in the hippocampus of patients with temporal lobe epilepsy during seizure activity (Chugani et al., 1993). This is thought to represent a response to the increased energy demands associated with restoring ionic gradients, replenishing neurotransmitter vesicles, and compensating for cellular stress after ictal activity. However, the dynamic response of glucose utilization associated with seizure activity is much more complicated than this simplistic coupling.

### Clinical Perspective

Altered glucose utilization occurs in patients with epilepsy, with both hypo- and hyper-metabolism commonly seen. A recent retrospective study of over 500 PET scans of patients with intractable epilepsy found that approximately 6% showed significant hypermetabolism (Bansal et al., 2016). Similar increases in glucose utilization have been seen in various forms of epilepsy, including focal cortical dysplasia (Namer et al., 2014), Sturge-Weber Syndrome (Alkonyi et al., 2011), and continuous spikes and waves during sleep (De Tiege et al., 2004). The low incidence of hypermetabolism may reflect that the vast majority of human studies examine the interictal period, when seizure activity is not occurring. Hypometabolism is much more frequently reported in patients with epilepsy. Decreased FDG labeling in the temporal lobe is, in fact, a strong predictor of seizure control following resection of the temporal pole for intractable seizures (Radtke et al., 1993). While the mechanistic underpinning of hypometabolism is not known, many suspect that the loss of neurons associated with hippocampal sclerosis results in decreased energy demand. While this may be true, other studies suggest that hypometabolism may be prevalent in areas where sclerosis is minimal, such as hippocampal area CA3 (Vielhaber et al., 2003). Because of the variety of metabolic changes observed in patients with epilepsy, there have been some controversies regarding the value of metabolic imaging data.

The literature reporting seemingly incongruent results may reflect biological diversity, as studies using imaging technologies with higher spatial resolution suggest regionally heterogeneous metabolic activity in individual patients suffering from epilepsy. Recent work using FDG-PET combined with electrocorticography in patients before surgery for intractable epilepsy reports extremely complex changes. Areas of both hypo- and hyper-metabolism were seen, and these areas had variable spatial overlap with sites of seizure initiation (Jeong et al., 2017). This supports a complex spatiotemporal relationship between metabolism and seizure activity that can vary between patients, between brain regions in the same patient, and perhaps even between individual seizures in the same patient. Importantly, FDG-PET does not report on glycolytic activity directly, but rather indicates glucose uptake. Therefore, combining FDG-PET with other measures can help increase our understanding of glucose utilization.

Another set of commonly used, clinically available tools assay brain metabolic activity by measuring blood flow and blood oxygenation. In generalized spike-wave epilepsy, cerebral blood flow has been shown to increase prior to ictal activity and then decrease following the seizure (Diehl et al., 1998). Intraoperative monitoring of hemoglobin oxygenation in temporal lobe epilepsy suggests decreased cerebral perfusion prior to ictal activity, followed by a significant increase after the seizure ends (Zhao et al., 2007). BOLD signal derived from fMRI, which also monitors hemoglobin oxygenation status, suggests that metabolic changes precede seizure activity, with the pattern of activity with respect to the seizure focus varying widely from patient to patient (Federico et al., 2005). When these measures of cerebral blood flow and oxygenation are combined with the FDG-PET findings discussed earlier, a picture begins to emerge regarding glucose utilization and the epileptic brain. During ictal activity, glucose utilization is acutely increased. Interictally, metabolic activity can be regionally increased or decreased, and likely reflects complex pathophysiology including cell loss, altered neuronal activity, and inflammation (Vezzani et al., 2015), all of which lead to regionally specific changes in metabolic activity. Because of the limitations of interrogating the human brain, preclinical studies have also been utilized to better understand the metabolic changes associated with seizure activity.

### Preclinical Perspective

Many of the clinical findings discussed above have been replicated in animal models. Glucose utilization is increased during experimental status epilepticus (SE) (Ingvar, 1986; Pereira de Vasconcelos et al., 2002) induced by bicuculline (Ingvar and Siesjo, 1983), pilocarpine, and kainic acid (Alvestad et al., 2008). Multiple studies also show subsequent hypometabolism, as measured with PDG-PET, beginning approximately 1–2 days after chemoconvulsant-induced status epilepticus and lasting as long as 42 days (Guo et al., 2009; Jupp et al., 2012). In fact, Jupp and colleagues showed that following kainic acid-induced status epilepticus, metabolic activity was decreased at 1 day post-SE, recovered slightly, and then decreased again during the chronic phase of epilepsy when spontaneous recurrent seizures appear (Jupp et al., 2012). This suggests that glucose consumption may fluctuate throughout the different phases of the epileptogenic process. Acute seizures lead to aberrant consumption of glucose to support cellular homeostasis. Quickly afterwards, glucose utilization is decreased during the post-ictal phase. Later, cellular- and network-level changes which contribute to the epileptogenic process result in varied metabolic needs. The dynamic nature of glucose utilization described here suggests that therapies targeting metabolism may have discrete windows of efficacy, paralleling the complex changes of metabolism observed in patients with epilepsy.

As with clinical studies, however, many of these assays monitor glucose uptake as a measure of metabolic activity. In the brain, glucose utilization is intimately linked with astrocytic metabolism, where astrocytes can mobilize glucose from glycogen stores or perform glycolysis to provide lactate to neurons for further metabolism. In a rat model of sustained seizures, glycogen concentrations were diminished after 20 min and 2 h of status epilepticus. Interestingly, lactate levels were elevated after 20 min of SE, but were decreased after 2 h of SE (Folbergrova et al., 1981). This suggests that seizure activity first results in glycogen store utilization to provide fuel in the form of lactate, but that the glycogen reserves are not sufficient to provide continuous fuel during a long period of SE. In a neonatal flurothyl seizure model, seizures induced an acute decrease in brain glucose levels (as measured by labeled carbon tracing) and a significant increase in lactate levels, although glycogen levels were stable (McDonald and Borges, 2017). This suggests that while glucose utilization during a seizure may be a global phenomenon, the source of glucose and how it is utilized may vary with age and experimental model (Wasterlain et al., 2010). Further complicating the picture, glucose may be directed to different metabolic processes depending on the oxygenation level. Glucose can provide energy via glycolysis under hypoxic or normoxic conditions, but once converted to pyruvate, further metabolism through oxidative phosphorylation requires oxygen, and thus requires normoxic conditions. In a canine model of epilepsy, MR spectroscopy was used to analyze brain metabolism postictally. Glycolytic end products appeared to increase gradually during seizures due to evolving anaerobic conditions during the ictal activity (Neppl et al., 2001). The relationship between oxygenation state and glycolytic activity, therefore, may contribute to the varying reports of glucose utilization in the epileptic brain. For example, if oxygen levels are low, glycolysis may be relied upon more heavily than oxidative phosphorylation. However, because glycolysis produces fewer ATP molecules than oxidative phosphorylation per glucose molecule, this may explain the increased need for glucose uptake.

Preclinical models have also demonstrated that different types of seizures can lead to different metabolic changes. Using combined NADH and FAD^+^ imaging alongside recording of oxygen levels, Ivanov et al. (2015) demonstrated unique metabolic changes in two *in vitro* models of seizure-like activity. In a zero-magnesium model, oxidative phosphorylation increased strongly during ictal activity and then decreased abruptly before the ictal activity ceased. Using a bicuculline model, oxidative phosphorylation increased in brief transients and then decreased during ictal activity (Ivanov et al., 2015). This study also suggests that glycolysis precedes epileptiform activity, as NADH production occurs without oxygen utilization. In various *in vivo* models, we also observe different seizure subtypes utilizing different energy supplies. In a mouse model of flurothyl-induced seizures, labeled glucose was used to track cerebral metabolic flux. Surprisingly, labeled glucose was not converted into labeled lactate, even though total lactate levels rose. This suggests that during a seizure, lactate does not arise directly from glucose, but rather from utilization of glycogen stores. In this study, McDonald and Borges also reported a significant inhibition of pyruvate dehydrogenase (PDH), the enzyme which converts pyruvate into acetyl-CoA for entry into the TCA cycle (McDonald and Borges, 2017). This suggests that selective suppression of glucose-derived substrates from entering into oxidative phosphorylation occurs in this model of acute seizures, perhaps as a mechanism to reduce oxidative stress. Conversely, in a study examining how kainic acid-induced seizures affect cerebral glucose utilization (Walls et al., 2014), the authors reported that both a sub-convulsive and convulsive dose of kainic acid led to an immediate increase in lactate levels. Interestingly, when a sub-convulsive dose was examined, astrocytic TCA cycle activity was increased but neuronal activity remained stable. When a convulsive dose was given, both neuronal and astrocytic metabolism were affected. This supports the idea that neuronal metabolic activity is preferentially enriched in states of energy crisis, whereas astrocytic metabolism is able to compensate in baseline or less-demanding activity states.

Throughout the examples described above, it is apparent that the metabolic changes associated with seizure activity are diverse, complicated, and variable depending on seizure type, regional focus, and individual patient. Therefore, in developing a metabolic therapy, it will be important to stratify patients based on the pathophysiologic processes contributing to their epilepsy. We will now turn our attention to the metabolic changes associated with TBI, as patients who suffer from TBI are at increased risk of epileptogenesis. Because of the latent period between the injury and onset of seizures, this patient population represents a unique opportunity to develop a metabolic therapy targeted to the epileptogenic process.

## Glucose Metabolism Is Dysregulated Following Traumatic Brain Injury

Traumatic brain injury occurs when a mechanical force acts on the brain and disrupts normal function. TBI encompasses a diverse range of injury types, severities, and patient demographics, and results in ≈2.8 million emergency room visits per year in the United States. Importantly, TBI can result in long-term neurological complications which affect patient quality-of-life, including neurodegeneration, behavioral dysfunction, and epilepsy. However, the heterogeneity of TBI in humans has made it difficult to fully understand the pathophysiology of these long-term neurological disorders. As we have already discussed how metabolism is disrupted with changes in activity state and in hyperexcitable/seizure-prone neuronal networks, changes in glucose utilization may provide a clue as to how complications emerge following injury (**Figure 4**). The acceleration/deceleration and shearing forces associated with TBI disrupt cellular membranes and result in massive neurotransmitter release, blood-brain-barrier compromise (Shlosberg et al., 2010), and loss of the electrochemical gradients required for neuronal signaling (reviewed in Prins et al., 2013; Dulla et al., 2016). Thus, during the acute period after injury, there is a significant change in energetic needs for the regions affected. Unfortunately, our knowledge of how these molecular and cellular insults translate into changes in energy utilization is limited. Here, we review the multiple clinical and preclinical studies performed to gain a better understanding of how glucose utilization changes to compensate for this pathology.

### Clinical Perspective

Brain imaging in humans following TBI, including PET scanning and magnetic resonance spectroscopy (Stovell et al., 2017), can reveal changes in glucose uptake/utilization. A study in 1997 examined 28 patients who suffered from a severe TBI, and found evidence of increased cerebral glucose utilization in the majority of patients within the first week following injury (Bergsneider et al., 1997). This study also revealed that some patients had increased glucose uptake in focal regions (particularly when associated with a focal mass lesion TBI), while others had increased uptake globally throughout the cortex. Later work revealed that global increases in glucose utilization are more common in severely injured TBI patients, relative to mild TBI patients (Bergsneider et al., 2000). When specifically examining the white matter after moderate to severe TBI, patients exhibited a decrease in oxygen utilization without a decrease in glucose utilization (Wu et al., 2004a), suggesting a possible uncoupling of aerobic and anaerobic fuel utilization, as well as providing evidence for metabolic disruption in the setting of diffuse subcortical white matter injury. Patients with lower extracellular glucose and higher extracellular glutamate levels within the first week following injury, findings possibly associated with early hyperglycolysis and indicative of dysregulated glucose utilization, had worsened outcomes at 6 months post-injury (Vespa et al., 2003). This finding suggests a crucial role for metabolic and activity state changes in long-term patient outcomes. Importantly, acute metabolic changes may not be uniform across patients as some studies have observed decreased glucose utilization in gray matter acutely following injury (Wu et al., 2004b). This underscores the importance of developing approaches to rapidly and quantitatively assay brain metabolic function so that patients with different injury types, severities, and timelines may be compared appropriately.

Following the initial stage of focal or global hypermetabolism, there is significant evidence of long-term hypometabolism across multiple brain regions in the months to years following TBI. As early as several days following injury, there is a decrease in cerebral blood flow and oxygen utilization in the peri-lesional area (Kawai et al., 2008). However, even when studied >1 year following diffuse axonal injury, there is decreased cerebral oxygen utilization in 60% of patients (Shiga et al., 2006). In fact, cerebral hypometabolism may be a prognostic indicator of long-term outcomes following TBI, as it is for outcomes following surgical resection in temporal lobe epilepsy (Sakamoto et al., 2009). There is a reported relationship between the degree of hypometabolism and the level of patient consciousness after TBI, where patients in a vegetative state had more widespread, reduced metabolic flux relative to patients with higher levels of function (Nakayama et al., 2006). In fact, even within the acute period (first 5 days post-TBI), decreased glucose utilization in specific brain regions (thalamus, brainstem, and cerebellum) also correlated with patients’ levels of consciousness (Hattori et al., 2003). Additionally, cerebral metabolism following TBI positively correlates with full-scale IQ scores (Kato et al., 2007), suggesting that decreased cerebral fuel utilization is associated with worsened cognitive and behavioral outcomes.

It is important to clarify that the findings of altered glucose uptake or oxygen utilization on imaging are not easily interpretable as changes in glycolysis/glucose utilization. Studies using microdialysis of metabolites, or tracking 13C-labeled carbon from glucose through to its products (Carpenter et al., 2014), can help us better understand how glucose metabolism is altered following injury. When 13C was followed, Dusick and co-authors found a preferential increase in glucose flux into the pentose phosphate pathway, particularly in the first 48 h after injury (Dusick et al., 2007). This finding argues against the traditional assumption that increased glucose uptake on PET scans indicates cerebral hyperglycolysis, although Jalloh and colleagues found increases in both glycolytic lactate production and flux through the pentose phosphate pathway after TBI (Jalloh et al., 2015). Examination of lactate (product of anaerobic glycolysis) and pyruvate (product of aerobic glycolysis to be utilized in the TCA cycle) from intrasurgical microdialysis showed increases in both of these products with increased glucose uptake on PET scan (Hutchinson et al., 2009). No change in the lactate:pyruvate ratio in this study suggested that glucose utilization by both aerobic and anaerobic glycolysis was increased following injury. Another microdialysis study in conjunction with intracortical depth EEG found an increased lactate:pyruvate ratio specifically during seizures or periodic discharges after injury, suggesting transient metabolic crises during periods of altered brain activity (Vespa et al., 2016) and preferential increases in anaerobic glycolysis during seizures, as described above (Neppl et al., 2001).

While these human studies clearly reveal long-term dysregulation of glucose metabolism following TBI, there are significant limitations in interpretation due to the inability to clearly track individual metabolic processes in brain tissue as well as the heterogeneity of patient injuries and timeline of study participation. The use of preclinical animal models has provided additional understanding of post-TBI pathophysiology by standardizing injury mechanism, location, and timeline (Guglielmetti et al., 2017; Van Horn et al., 2017).

### Preclinical Perspective

There are multiple animal models of TBI used for preclinical experimentation, including controlled cortical impact (CCI), fluid percussion injury (FPI), and weight drop. Each of these models recapitulates aspects of human TBI, including cellular losses (Buritica et al., 2009; Cantu et al., 2014), behavioral dysfunction (Fox et al., 1999; Chauhan et al., 2010; Zhao et al., 2012), and post-traumatic epilepsy (Bolkvadze and Pitkanen, 2012; Bolkvadze et al., 2016), while allowing experimental control of injury type, location, and severity across subjects. Newer models of closed-head and repetitive brain injury may more closely resemble the injury mechanisms experienced by most human patients, although these have not been as extensively studied as CCI, FPI, and weight drop. Each of these preclinical models can produce different pathological outcomes, based on variables such as injury severity and location. Drawing conclusions based on data from multiple models may provide better understanding of TBI pathophysiology and more fully represent the diversity of real-world human brain injury.

Some animal models have recapitulated the early hypermetabolism and prolonged hypometabolism observed in humans. For example, after FPI, there are local increases in glucose utilization at early time points (Andersen and Marmarou, 1992), which then transition to a hypometabolic period that resolves to baseline by several days following injury (Yoshino et al., 1991). Studies in juvenile rats follow this pattern (Thomas et al., 2000; Casey et al., 2008), but suggest that the metabolically depressed period may resolve faster in young animals or that the injury-induced energy crisis may be delayed in young relative to adult animals (Deng-Bryant et al., 2011). This phenomenon could begin to explain the resilience and relative neuroprotection of juveniles to TBI. As late as 3 months following mild TBI (weight drop) in rats, there are observable regionally specific changes in glucose uptake (Vallez Garcia et al., 2016). As observed in humans, long-term hypometabolism is associated with worsened outcomes in animal models. A composite score of serial PET scans from 1 week, 1 month, and 3 months after rat FPI revealed that hypometabolism in the hippocampus ipsilateral to injury was more significant in epileptic versus non-epileptic animals (Shultz et al., 2013). Interestingly, changes in glucose uptake in the acute, sub-acute, and chronic period are all associated with underlying regional pathophysiology such as reactive astrocytosis and microgliosis (Brabazon et al., 2017). This association may provide a cellular-level explanation for the correlation between glucose metabolism and functional outcome, and may provide insight into possible human biomarkers or therapeutic targets.

Interestingly, the use of excitatory neurotransmitter antagonists, particularly APV to block NMDA receptors, prevented the increase in glucose utilization after FPI (Kawamata et al., 1992), suggesting that the metabolic changes after injury are in fact activity-dependent. There is also evidence that excessive activation during a vulnerable period after TBI results in worsened outcomes, as motor cortex stimulation 1 day after injury causes increased cortical degeneration (Ip et al., 2003). Additionally, a repeated brain injury during a vulnerable metabolic period (such as 24 h after a mild TBI) can lead to functional impairment, increased lesion volume, and increased reactive astrocytosis relative to repeated injury during a less vulnerable time (15 days after TBI) (Selwyn et al., 2016). When the brain is already in a state of energy crisis, additional aberrant activation creates further energetic demands and can cause cell death, excitotoxicity, and ultimately worsened outcomes.

A key advantage of utilizing animal models is the ability to harvest brain tissue after injury to assess changes in expression patterns. These changes provide insight into which processes are up- or down-regulated following injury. Examination of the transcriptome after CCI in mice shows increased expression of glycolytic enzymes at 6 h after injury, with later decreases in glycolytic enzyme expression at later time points (Zhou et al., 2017). Similarly, severe FPI in rats resulted in increased expression and enzymatic activity of proteins involved in glycolysis at early time points after injury (Amorini et al., 2016). This study also found a slight delay in the hyperglycolytic response in animals with mild injury relative to severe injury. Together, these findings strongly support dynamic regulation of glycolytic activity following TBI in animal models.

Additionally, animal studies can begin to elucidate the cell type-specific changes of metabolism after injury. For example, GLUT3 expression (which is responsible for glucose transport into neurons) is increased by 300% from 4 to 48 h after injury, while glial transporter GLUT1 is not changed (Hamlin et al., 2001). This finding suggests that neuronal glucose uptake and utilization is preferentially increased in the post-injury period, relative to astrocytic glucose uptake. By 14 days after injury, the oxidative metabolism of glucose is decreased preferentially in neurons but not astrocytes (Bartnik-Olson et al., 2010).

In addition to helping to identify the basic pathophysiology after TBI, preclinical models also allow for the study of possible therapeutic approaches. One example is the delivery of glycolytic end-product pyruvate to animals following injury, which has been shown to attenuate posttraumatic hypometabolism, be neuroprotective (Moro et al., 2016), and ameliorate deficits in working memory (Moro et al., 2011). The ketogenic diet has also been investigated as a therapeutic approach following TBI (reviewed in Prins and Matsumoto, 2014), and has been shown to decrease edema, cytochrome *c* release, Bax upregulation, and apoptotic cell death after weight drop in rats (Hu et al., 2009a,b).

As we begin to better understand the etiology of post-TBI complications, we can identify relevant biomarkers to assess the efficacy of various therapeutic approaches. As of now, there are no clear biomarkers that indicate risk for post-traumatic epileptogenesis after TBI. In the preclinical models discussed above, only some are associated with post-traumatic epilepsy. Further, within each model, only a small portion of rodents develop spontaneous electrographic and behavioral seizure activity while the others remain non-epileptic. This resembles human TBI, where depending on injury severity, only a subset of patients will go on to develop PTE. It is not well-understood why some animals (and humans) are more vulnerable than others to PTE, and without good biomarkers for epileptogenesis to stratify study subjects, it is difficult to power a preclinical drug study with our available models. However, since glucose metabolism is clearly dysregulated in both epileptic brains and following TBI, this may be an exciting area to look for possible biomarkers. Additionally, attempts to maintain physiologic glucose utilization (avoiding acute hyperglycolytic and chronic hypometabolic states) may constitute a valuable therapeutic approach that should be investigated.

## A Role for Glycolytic Inhibition in Modulating Aberrant Network Function

As described above, brain metabolism (particularly glucose utilization and glycolysis) plays a key role in maintaining network activity in both physiologic and pathologic states. There has been increasing interest in manipulating brain metabolism in order to control aberrant activity states. The rationale behind this approach is well-founded, based on the clinical success of the ketogenic diet (KD) in humans suffering from intractable epilepsy. This diet is very low in carbohydrates and high in fats, which is thought to cause a shift in brain fuel utilization from glucose/glycolysis to ketone bodies/ketosis. Many molecular mechanisms have been proposed to modulate the antiepileptic effects of the KD, in an effort to find a small molecule mimic [reviewed in (Yellen, 2008; Milder and Patel, 2012; Boison, 2017; Rho, 2017; Simeone et al., 2018) and others]. Additionally, the KD is now being utilized for other neurological conditions, including neurodegenerative diseases like amyotrophic lateral sclerosis, Alzheimer’s disease, and Parkinson’s disease (reviewed in Paoli et al., 2014), and psychiatric conditions (Bostock et al., 2017), although with limited evidence in humans in the latter case. Interestingly, the KD is also under investigation for treating cancer, where controlling metabolic substrate supply to the most metabolically active cancer cells may be a useful therapy.

The ketogenic diet is often difficult for patients to maintain, as the meal options are limited and sometimes difficult to access. Thus, how can we mimic the benefits of the KD with a pharmacological tool? Some studies have utilized β-hydroxybutyrate, which is one of the main ketone bodies upregulated in KD. Exposure to β-hydroxybutyrate in brain slices reduces glucose utilization and stimulates pyruvate consumption (Valdebenito et al., 2016) suggesting a shift away from glycolysis. Ketone bodies have also been shown to effectively reduce hyperexcitability and seizures in multiple different *in vitro* and *in vivo* models (reviewed in Simeone et al., 2018). On the other hand, a study in seizure-prone EL mice (Meidenbauer et al., 2011) showed that decreased glucose utilization (with decreased glucose in the diet, with or without the glycolytic inhibitor 2-DG) is required to replicate the protective effects of the KD, while β-hydroxybutyrate supplementation was not sufficient to protect against seizures (Meidenbauer and Roberts, 2014).

**FIGURE 3:**
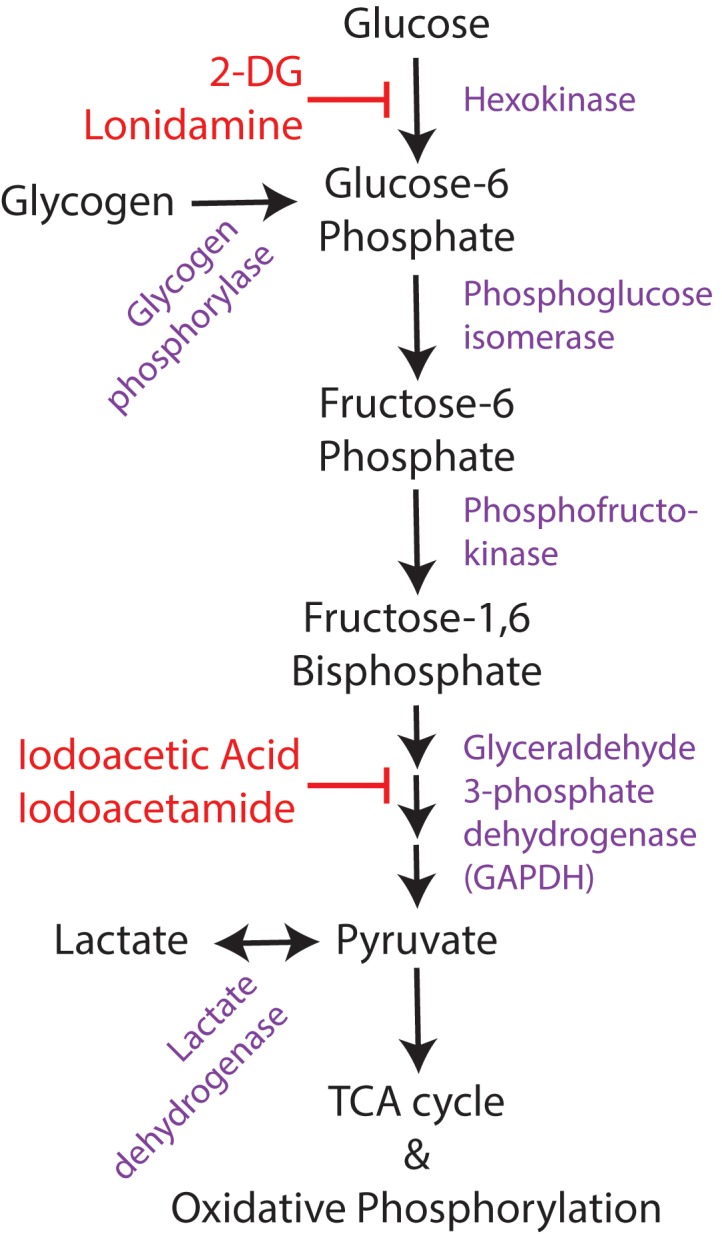
Overview of glucose utilization. Simplified schematic of glucose utilization, focusing on the steps of the glycolytic pathway. Enzymes shown in purple and pharmacological inhibitors in red. 2-DG, 2-deoxyglucose.

Direct inhibition of glycolysis has been extensively studied as an anticonvulsant target, due to the known relationship between glucose utilization and neuronal excitability and activity. While there are multiple methods to inhibit glycolysis (**Figure 3**), including pharmacologic hexokinase inhibition with lonidamine (Ghosh et al., 2016), hexokinase feedback inhibition with its end-product glucose 6-phosphate (Liu et al., 1999), glyceraldehyde-3-phosphate dehydrogenase (GAPDH) inhibition with iodoacetic acid or iodoacetamide (Schmidt and Dringen, 2009), and genetic manipulations to knock down enzymatic activity at different stages of glycolysis (Wu et al., 2018), the most commonly used method is 2-deoxyglucose. 2-deoxyglucose (2-DG) is a glucose analog that competitively inhibits glycolysis at the rate-limiting enzyme hexokinase. The end-product of 2-DG’s interaction with hexokinase (2-deoxy-glucose-6-phosphate), lacks the hydroxyl group required for the action of the next glycolytic enzyme, phosphoglucose isomerase, so glycolysis is halted. 2-DG is already in use clinically as a chemotherapeutic agent for cancer (Zhang et al., 2014), as neoplastic cells have increased glycolytic activity via the Warburg effect. 2-DG is well-tolerated in humans (Ockuly et al., 2012; Zhang et al., 2014) and is continuing to be investigated as a therapeutic adjuvant with other cancer treatments.

2-DG has been shown to have anticonvulsant properties in many different slice and *in vivo* models of epilepsy (reviewed in Stafstrom et al., 2008). In key work by Stafstrom et al. (2009), the authors showed a decrease in interictal epileptiform bursts in hippocampal slices treated with 7.5 mM [K^+^], 4-aminopyridine (4-AP), or bicuculline (all *in vitro* models of hyperexcitability). They showed an increase in the after-discharge threshold in the perforant path kindling model with 2-DG (also shown by Garriga-Canut et al., 2006), suggesting antiepileptic action. As more evidence *in vivo*, 2-DG has been shown to decrease seizure severity and duration following pilocarpine (Lian et al., 2007) and to increase the seizure threshold in the 6-Hz corneal stimulation model (Gasior et al., 2010). More recently, bath-applied 2-DG has been shown to suppress spontaneous neuronal firing and zero-magnesium-induced epileptiform bursts in hippocampal slices, while intracellular application of 2-DG to a single neuron is not sufficient to reduce its spontaneous firing (Shao and Stafstrom, 2017). This study suggests a network effect of glycolytic inhibition beyond what is capable by controlling a single neuron’s activity. Taken together, these results strongly support the role of 2-DG as an anticonvulsant target.

While there have been reports of 2-DG decreasing seizure threshold or initiating epileptogenesis (Gasior et al., 2010; Samokhina et al., 2017), these findings may be attributable to the fact that chronic hypometabolism or severe glucose deprivation can independently cause seizures. Also, several clinically effective anticonvulsants have mixed effects in animal models (Loscher, 2009), depending on the exact mechanism of action of the drug. Further, a new study by Nedergaard shows that the proconvulsant effect of 2-DG can be reproduced by blocking oxidative phosphorylation (Nedergaard and Andreasen, 2018), providing a possible mechanism to describe ictal facilitation by 2-DG that is distinct from the anticonvulsant effects of glycolytic inhibition. Thus, the varied results of 2-DG do not rule out glycolysis as a key anticonvulsant target.

There have been several mechanisms of action proposed for the anticonvulsant effects of 2-DG. First, 2-DG can directly suppress synaptic transmission, hyperpolarize neurons, and decrease membrane resistance (Zhao et al., 1997). This study implicated an adenosine-dependent increase in potassium conductance as a possible mechanism for the change in neuronal membrane properties. A study from 2016 identified an additional bicuculline-sensitive tonic current with 2-DG application, suggesting a role for the potentiation of GABAergic conductances as an additional mechanism for 2-DG’s anticonvulsant effects in the 4-AP slice model of hyperexcitability (Forte et al., 2016). In addition to its direct effects on neuronal excitability, 2-DG has also been reported to alter transcriptional activity. For example, 2-DG has been shown to attenuate electrical kindling-induced upregulation of brain-derived neurotrophic factor (BDNF) through an NRSF-dependent mechanism (Garriga-Canut et al., 2006). 2-DG has also been shown to increase expression of K*_ATP_* channel subunits Kir6.1 and Kir6.2 following pilocarpine (Yang et al., 2013), which could explain the increased potassium conductance and anticonvulsant effect of the treatment. Thus, 2-DG is likely acting through rapid, reversible effects on neuronal excitability as well as through longer-term mechanisms affecting neuronal expression profiles.

Other metabolic molecular targets that shift neuronal metabolism away from glycolysis have been investigated for their anticonvulsant properties, including lactate dehydrogenase (LDH), BAD/K*_ATP_* channels, and fructose-1,6-diphosphate. LDH is a component of the ANLS, which allows for the conversion of lactate to pyruvate after delivery to neurons. Using an anticonvulsant screen, it was found that clinical anticonvulsant stiripentol exerted its effects through LDH inhibition, which hyperpolarized neurons and could be counteracted with delivery of glycolytic end product pyruvate (Sada et al., 2015). The Yellen group has explored a K*_ATP_*-channel-dependent mechanism through which the BAD knockout mouse is resistant to picrotoxin-induced epileptiform activity. Specifically, they found that the protective effect of BAD knockout (a protein which is involved in apoptosis and glucose metabolism) in reducing epileptiform activity is abolished through genetic or pharmacologic blockade of K*_ATP_* channels (Martinez-Francois et al., 2018; Yellen, 2018). Finally, fructose-1,6-diphosphate, which is thought to shift glucose utilization away from glycolysis and toward the pentose phosphate pathway (Stringer and Xu, 2008), has been shown to be anticonvulsant in multiple rat models of epilepsy (Lian et al., 2007) and protective against epileptogenesis in an amygdaloid-kindling seizure model in rats (Ding et al., 2013).

## Can Inhibiting Glycolysis Reduce Post-Traumatic Epilepsy Following Traumatic Brain Injury?

### Broad Spectrum Intervention in a Complex Environment

To date, no clinical treatments exist which can reduce the incidence of post-traumatic epilepsy following TBI. Anticonvulsants (such as phenytoin and levetiracetam) and glucocorticoids have been examined in clinical trials to prevent PTE, but have not shown significant long-term beneficial effects (Chang et al., 2003; Trinka and Brigo, 2014; Kirmani et al., 2016). Could metabolic therapies provide a novel way forward? As described above, preclinical studies support the use of glycolytic inhibition to reduce seizures in multiple models. These studies, however, draw on 2-deoxyglucose’s *anticonvulsant* effects and have not explored the possible disease-modifying, anti-epileptogenic effects of the treatment. In addition to 2-DG’s anticonvulsant effects, glycolytic inhibition following TBI may result in other benefits that prevent the development of post-traumatic epilepsy. Metabolic manipulation as a therapeutic target is a rational approach based on the body of evidence presented above. First, diverse and robust metabolic changes occur following TBI. At a basic level, this implicates metabolism as a part of disease pathology, and therefore a potential avenue for therapeutic intervention. Second, inhibiting metabolic systems offers the opportunity to affect a wide range of energy-dependent processes that may contribute to disease pathology. Neurotransmission, maintenance of ionic gradients, and inflammatory processes (among others) all have significant energy demands and may contribute to disease progression. While disrupting metabolism may lack specificity, its breadth of effects may contribute to a truly disease-modifying approach. Additionally, this approach may be more specific than first appreciated, as it aims to decrease the activity of only the most active cells. Third, increased metabolic activity acutely following brain injury may drive secondary injury processes that contribute to epileptogenesis. To date, no data exists to support or refute a direct link between them, but a deeper understanding of how different pathophysiological processes interact to result in post-traumatic epileptogenesis will help drive novel therapeutic approaches. If intervening in metabolic pathways after injury is protective, it is likely not via acute anticonvulsant effects, as other traditional anticonvulsant drugs have failed to prevent PTE. Instead, it may be acting by preventing other epileptogenic processes. Finally, manipulating metabolism, in some ways, is reminiscent of TBI treatment with hypothermia. While hypothermia has many effects, it slows metabolic activity via its effects on enzyme kinetics. A recent meta-analysis of therapeutic hypothermia for TBI suggests it may improve neurological outcomes for adults following focal TBI (Crompton et al., 2017; but see also Maas and Stocchetti, 2011; Andrews et al., 2016). Because the mechanistic rationale is strong and the need for disease-modifying therapies to prevent PTE is great, it makes sense to consider how we might harness 2-DG (or other strategies to inhibit glycolysis) to improve patient outcomes following TBI.

### Metabolic Intervention Considerations: Timing May Be Essential

**FIGURE 4:**
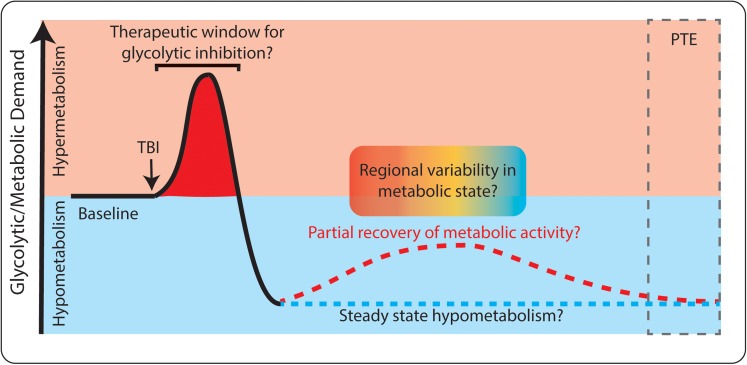
Dynamic glucose utilization following TBI. Both hyper-metabolism (red) and hypo-metabolism (blue) occur following TBI. Initially, glucose utilization increases, which may be a target for therapeutic intervention. Later, hypometabolism occurs and long-term complications such as post-traumatic epilepsy (PTE) can develop. Both consistent hypometabolism (blue dashed line) and partial recovery of hypometabolism (red dashed line) have been reported following TBI. There may also be spatially specific changes in metabolism within individual patients (red/blue gradient).

Based on the existing evidence, attenuating glycolysis may only be advantageous during the first hours to days following TBI (as depicted in **Figure 4**). Clinical literature supports that TBI-induced hypermetabolism occurs only in the short term after TBI. The injury itself causes cells to rupture and release their intracellular contents (including pro-excitatory species like glutamate), breaches the blood-brain barrier, activates immune and inflammatory cascades, and reduces local blood flow and oxygenation. All of these events could favor the use of glycolysis to power neuronal activity and inflammatory activity, especially in a local low-oxygen environment. If any of these early events, and their immediate downstream effects, contribute to the development of PTE and rely on increased glycolysis, then attenuating excessive glycolytic activity may be beneficial. The time window in which hypermetabolism occurs may be extremely short, perhaps lasting only minutes to hours following injury (Kawamata et al., 1992; Prins et al., 2013; Gajavelli et al., 2015). Once the initial hypermetabolic state is resolved, the brief window of opportunity to provide metabolic intervention is likely lost. As outlined above, a period of hypometabolism occurs in humans and animal models in the days to months following TBI. During this phase, there would no longer be rationale to inhibit glycolysis, as we would not want to further reduce metabolic activity.

### What Pathological Events Following TBI Could Be Targeted to Avoid PTE?

There are surprisingly few risk factors identified that clearly correlate with an increased risk of developing PTE following TBI. The only clear prognostic indicators are the severity of the injury and age at time of injury (Annegers et al., 1998; Ritter et al., 2016; Piccenna et al., 2017). How do we know which aspects of TBI pathophysiology should be targeted to reduce PTE? Recent preclinical studies have identified hippocampal shape changes visualized using MRI that reliably predict the development of PTE in the rat fluid percussion model of TBI (Liu et al., 2010). Excitingly, a recent study showed that increased levels of IL-1β, a proinflammatory cytokine, was associated with increased risk of PTE in humans (Diamond et al., 2014). While this finding has not yet been developed into an established biomarker and may have caveats, it is an exciting step forward. Perhaps the most interesting development in identifying PTE risk factors is a recent study examining EEG abnormalities seen in the intensive care unit following TBI in humans. Kim and colleagues reported that increased epileptiform abnormalities (including spikes and sharp waves, periodic epileptiform discharges, and rhythmic activity) in the days following TBI indicate elevated risk for developing PTE (Kim et al., 2018).

Deeper mechanistic insights to link TBI and PTE are still sorely lacking, but will be crucial in developing new biomarkers and therapeutic approaches. For example, does reducing the amount of tissue lost following TBI protect against PTE? What circuit-level changes drive epileptiform activity and epileptogenesis post-injury? How do secondary injury mechanisms, including inflammation, oxidative damage, and apoptosis, contribute to epileptogenesis? These mechanistic questions cannot be easily addressed using clinical studies due to the heterogeneity of human TBI and the ethical and technical limitations of utilizing human subjects. Therefore, we rely on preclinical data to guide our development of translatable clinical approaches. A dizzying array of cellular and molecular changes occur following TBI and may contribute to the development of PTE. Here, we will discuss specific targets which we suspect are especially relevant to epileptogenesis, and will propose how glycolytic inhibitors could be utilized to attenuate these processes.

#### Tissue Loss

Because injury severity is related to the risk of post-traumatic epilepsy, it stands to reason that reducing gross and cellular tissue loss after TBI may improve outcomes following injury. Tissue loss can arise from the primary injury (Hemphill et al., 2015) and from secondary injury mechanisms that take place later. By the time a patient presents clinically, the initial mechanical force has already occurred and the primary injury cannot be undone. However, evidence supports that much of the tissue loss in TBI occurs due to the second wave of neuronal injury caused by excitotoxicity and ischemic insult (McIntosh et al., 1998; Gasior et al., 2006). Thus, translatable work should focus on ameliorating secondary injury, as the therapeutic timeline is more clinically feasible. Because 2-DG can acutely decrease hyperexcitability, it may also be able to reduce excitotoxicity. On the other hand, ongoing glucose metabolism provides energy to the system, which may be required to maintain cell health. Perhaps reducing glycolysis will favor glucose flux through the pentose phosphate pathway, which supports replenishment of the antioxidant glutathione (by providing NADPH) and may be protective following injury. Thus, the goal would not be to completely shut down glucose utilization or to starve the tissue, but instead to direct metabolism toward processes which will maximize tissue health. Direct measures of how inhibition of glycolysis may prevent excitotoxicity and cellular loss are sparse, requiring further study to determine if this approach may ameliorate secondary injury and reduce lesion volume after injury. This avenue may prove fruitful, however, as the ketogentic diet has been shown to result in reduced cortical tissue loss following CCI (Prins et al., 2005; Appelberg et al., 2009).

#### Inflammation

Traumatic brain injury induces significant and widespread neuroinflammation (Corps et al., 2015; Gadani et al., 2015) which is thought to be involved in the development and progression of epilepsy (Vezzani et al., 2011). Neuroinflammation following injury can include the activation of microglia and astrocytes, invasion of peripheral immune cells, and upregulation of inflammatory signaling cascades. As mentioned above, the presence of inflammatory molecules in the CSF following TBI are indicative of increased risk for developing PTE. Are any of these systems potential targets for metabolic interventions? Interestingly, inhibiting glycolysis using 2-DG led to the death of microglia in culture but a significant increase in neuronal cell loss following hypoxic insult (Vilalta and Brown, 2014). This suggests that large-scale inhibition of inflammation may not be beneficial following brain insult. *In vivo* manipulations, which are likely more relevant to human TBI, do support using metabolic approaches to minimize inflammation following brain insult. Caloric restriction was shown to reduce microglial activation in the cortical stab model of TBI (Loncarevic-Vasiljkovic et al., 2012), although caloric restriction occurred before the injury in this experiment. Studies examining the effects of the ketogenic diet on neuroinflammation, however, were not conclusive (Gasior et al., 2006). Interestingly, inhibiting glycolysis with 2-DG has been shown to reduce disease progression and inflammatory cell infiltration in models of CNS autoimmune disorders (Liu et al., 2018) and in experimental activation of inflammatory signaling in the brain (Tannahill et al., 2013), suggesting a possible neuroprotective role for this approach.

#### Synaptic Transmission

From a simplistic viewpoint, the development of PTE is likely due to increased neuronal excitation and decreased neuronal inhibition following TBI. A great deal of evidence exists for both changes following experimental TBI in animal models. A comprehensive analysis of these changes falls outside of the scope of this discussion, but are reviewed elsewhere (Hunt et al., 2013; Park and Biederer, 2013; Guerriero et al., 2015). Some specific examples of TBI-related changes in synaptic activity include loss of inhibitory interneurons, decreased synaptic inhibition, increased synaptic excitation, and exuberant growth of excitatory synaptic connections (Hunt et al., 2010, 2011; Cantu et al., 2014; Hsieh et al., 2017). Although preclinical studies have failed to demonstrate robust antiepileptogenic effects of the ketogenic diet (Schwartzkroin et al., 2010; McDougall et al., 2018), metabolic approaches may still have potential to restore normal synaptic function following TBI. For example, 2-DG treatment inhibits synaptic vesicle recycling (Ashrafi et al., 2017), thereby putting the brakes on synaptic activity during the metabolic crisis period following TBI. Additionally, inhibiting glycolysis (but not oxidative phosphorylation) reduces AP width and amplitude (Lujan et al., 2016), supporting a role for metabolic manipulation in controlling neuronal excitability and synaptic function. Finally, there is evidence supporting that different neuronal cell types utilize unique metabolic pathways to generate ATP (Kann et al., 2014). As compared to excitatory neurons, inhibitory interneurons contain more mitochondria (Gulyas et al., 2006), generate a different metabolic response to neuronal activity (Peng et al., 1994), and express a unique cadre of metabolic enzymes (Zeisel et al., 2015). The differences between energy utilization in different neuronal subtypes may enable cell type-specific metabolic manipulation of neuronal circuit activity through each cell’s unique sensitivity to glucose and/or oxygen deprivation. Thus, a metabolic approach may be able to preserve the inhibitory tone of the network by targeting aberrant activity in some neuron types, but not others.

### Caveats and Considerations

There are several conceptual concerns that arise when considering glycolytic inhibition as a treatment to prevent PTE following TBI. First, why would one consider reducing energy production in a brain that desperately needs energy to restore cellular and network function after injury? This becomes even more relevant when considering the long-term hypometabolic state that exists following TBI. In fact, there is significant controversy among clinicians as to whether glucose should actually be supplemented for post-TBI patients (Arlotta et al., 2008; Pecha et al., 2011; Zhao et al., 2011; Moro et al., 2013; Shijo et al., 2015; Shi et al., 2016). Additionally, work from Schallert and Hernandez supports the role for acute neuronal/network activity in behavioral recovery after lesional injury, as network inhibition with a short-term GABA agonist results in long-term impairment of behavioral recovery (Hernandez and Schallert, 1990). In response to this concern, it becomes clear that complete starvation or inhibition of metabolic activity is not helpful, particularly in the long term. Metabolic manipulation would likely be most effective in the acute phase, in the first few hours following TBI, and would focus on targeting aberrant, excessive glucose utilization to bring it back down to physiologic levels. Patient stratification using PET scanning or fMRI would help to target this therapy to the patients with the most severe hyperglycolysis, and would ensure that the patient had not already entered the hypometabolic state. Furthermore, the beneficial effects observed with the ketogenic diet may not be mediated by the inhibition of glycolysis, but may be through promoting ketosis (Prins and Matsumoto, 2014). Providing β-hydroxybutyrate, a ketone body metabolized through ketosis, increases ATP levels following TBI (Prins et al., 2004) and reduces tissue loss following focal ischemia (Suzuki et al., 2002), independent of glycolytic inhibition. This suggests that a combined approach of inhibiting glycolysis and supplementing ketosis may have the most therapeutic efficacy. It is also important to consider the role of oxidative stress after TBI. Oxygen supply is compromised after injury, and preclinical studies support the use of antioxidant treatments (such as *N*-acetylcysteine) to reduce tissue loss and improve behavioral function after TBI (Bhatti et al., 2017). Further studies are required to understand the relationship between glycolytic activity and oxidative stress in the injured brain. Finally, prolonged treatment with 2-DG may have significant adverse effects, including reported cardiovascular complications (Terse et al., 2016). Again, this finding would support a short time course of treatment with glycolytic inhibitors, only to target the increased glucose utilization in the first hours to days following injury.

## Conclusion

The challenges to treating TBI and preventing PTE are extensive. Heterogeneous injury categories, diverse metabolic responses to injury, and difficulty in powering both preclinical and clinical trials for PTE all make this a daunting translational problem. Based on the compelling evidence of metabolic changes following TBI, strong neuroprotective and anticonvulsant properties of the ketogenic diet, and multiple small molecule approaches to manipulate metabolism, we believe that therapeutic opportunities exist to harness metabolic systems to reduce PTE. There are many outstanding questions the field must address, including identifying the cellular sites and types of glucose utilization during normal brain function and after TBI, developing diagnostic tools that provide molecular insight into brain metabolism, understanding how metabolism contributes to post-traumatic epileptogenesis, and identifying biomarkers to stratify the patients at highest risk of developing PTE. We would recommend prioritizing the development of a quantitative assay to assess changes in glucose utilization in the brain following TBI. This assay could serve as both a prognostic and a predictive biomarker for PTE. Increased glucose utilization may be prognostic as it is likely associated with uncontrolled network activity, consistent with epileptogenesis. It may also serve as a predictive biomarker of which patients would respond best to a metabolically targeted therapy, such as glycolytic inhibition. This is especially important as hyper-glycolysis may only occur in a brief temporal window and/or in a subset of patients following TBI. Developing new tools and biomarkers is critical, as the clinical needs of TBI patients are clear: to improve long-term quality-of-life and to prevent devastating TBI complications such as PTE. Continued collaboration between basic scientists and clinicians will allow for better understanding of post-TBI pathophysiology and will ultimately advance novel interventional strategies to help these patients.

## Author Contributions

JK and CD conceived of and wrote the manuscript.

## Conflict of Interest Statement

The authors declare that the research was conducted in the absence of any commercial or financial relationships that could be construed as a potential conflict of interest.
